# Histone deacetylase inhibitors potentiate photodynamic therapy in colon cancer cells marked by chromatin-mediated epigenetic regulation of *CDKN1A*

**DOI:** 10.1186/s13148-017-0359-x

**Published:** 2017-06-08

**Authors:** Andrea Halaburková, Rastislav Jendželovský, Ján Kovaľ, Zdenko Herceg, Peter Fedoročko, Akram Ghantous

**Affiliations:** 1 Institute of Biology and Ecology, Faculty of Science, Pavol Jozef Šafárik University, Košice, Slovakia; 2 Epigenetics Group, International Agency for Research on Cancer (IARC), 150 Cours Albert Thomas, 69008 Lyon, France

**Keywords:** Histone deacetylase inhibitors, Hydroxamic acids, Short-chain fatty acids, Hypericin, Photodynamic therapy, Colorectal cancer, *CDKN1A*, Chromatin regulation

## Abstract

**Background:**

Hypericin-mediated photodynamic therapy (HY-PDT) has recently captured increased attention as an alternative minimally invasive anticancer treatment, although cancer cells may acquire resistance. Therefore, combination treatments may be necessary to enhance HY-PDT efficacy. Histone deacetylase inhibitors (HDACis) are often used in combination treatments due to their non-genotoxic properties and epigenetic potential to sensitize cells to external stimuli. Therefore, this study attempts for the first time to investigate the therapeutic effects of HDACis in combination with visible light-mediated PDT against cancer. Specifically, the colorectal cancer cell model was used due to its known resistance to HY-PDT.

**Results:**

Two chemical groups of HDACis were tested in combination with HY-PDT: the hydroxamic acids Saha and Trichostatin A, and the short-chain fatty acids valproic acid and sodium phenylbutyrate (NaPB), as inhibitors of all-class versus nuclear HDACs, respectively. The selected HDACis manifest a favorable clinical toxicity profile and showed similar potencies and mechanisms in intragroup comparisons but different biological effects in intergroup analyses. HDACi combination with HY-PDT significantly attenuated cancer cell resistance to treatment and caused the two HDACi groups to become similarly potent. However, the short-chain fatty acids, in combination with HY-PDT, showed increased selectivity towards inhibition of HDACs versus other key epigenetic enzymes, and NaPB induced the strongest expression of the otherwise silenced tumor suppressor *CDKN1A*, a hallmark gene for HDACi-mediated chromatin modulation. Epigenetic regulation of *CDKN1A* by NaPB was associated with histone acetylation at enhancer and promoter elements rather than histone or DNA methylation at those or other regulatory regions of this gene. Moreover, NaPB, compared to the other HDACis, caused milder effects on global histone acetylation, suggesting a more specific effect on *CDKN1A* chromatin architecture relative to global chromatin structure. The mechanism of NaPB + HY-PDT was *P53*-dependent and likely driven by the HY-PDT rather than the NaPB constituent.

**Conclusions:**

Our results show that HDACis potentiate the antitumor efficacy of HY-PDT in colorectal cancer cells, overcoming their resistance to this drug and epigenetically reactivating the expression of *CDKN1A*. Besides their therapeutic potential, hypericin and these HDACis are non-genotoxic constituents of dietary agents, hence, represent interesting targets for investigating mechanisms of dietary-based cancer prevention.

**Electronic supplementary material:**

The online version of this article (doi:10.1186/s13148-017-0359-x) contains supplementary material, which is available to authorized users.

## Background

The plant St. John’s wort (*Hypericum perforatum*) is widely used worldwide, and its botanical derivative, hypericin (HY) [1], demonstrates chemopreventive [2], chemotherapeutic, antiviral, and antidepressant [3] properties. Moreover, HY is a natural photoactive compound able to accumulate in cancer cells wherein it induces cell death upon activation by visible light [4, 5]. Photodynamic therapy (PDT) involves a combination of three agents: light (of specific wavelengths), oxygen, and a non-toxic photosensitizer, such as HY [4], and represents an alternative minimally invasive method for the treatment of malignant disorders. Although HY-mediated PDT (HY-PDT) exhibits considerable effectiveness compared to conventional therapies, cancer cells may acquire resistance to HY-PDT. Therefore, combination treatments, particularly with non-genotoxic drugs, may serve as a fourth agent to enhance HY-PDT efficacy. Histone deacetylase (HDAC) inhibitors (HDACis) have often been used in combination treatments due to their non-genotoxic epigenetic potential, and they inhibit HDAC-mediated deacetylation, causing hyperacetylation of histones with consequent decondensing of chromatin structure and re-expression of epigenetically silenced genes [6]. HDACis alter a small proportion (9%) of the entire genome [7], with preference to gene promoters [8]. Among the genes whose expression is highly coordinated by HDACi-mediated chromatin modulation is the inhibitor of cyclin-dependent kinases, *CDKN1A*, the expression of which can be induced within 2 h of HDACi treatment [8, 9]. We hypothesize that chromatin regulation by HDACis, particularly at the *CDKN1A* gene, could sensitize cancer cells to photochemical and photobiological processes induced by HY-PDT. In particular, we aimed to test the antitumor efficacy of HY-PDT and HDACi combination treatments on an in vitro model of colorectal cancer (CRC), as this cancer is known to be resistant to HY-PDT [10].

Different HDACis have been or are currently being evaluated for chemopreventive and chemotherapeutic purposes, alone or in combination with various treatments [11, 12]. In this study, we have tested the combination of HY-PDT with two chemical groups of HDACis: (a) the hydroxamic acids Saha and Trichostatin A (Tsa), which are inhibitors of all classes of HDACs, and (b) the short-chain fatty acids valproic acid (Vpa) and sodium phenylbutyrate (NaPB), which are inhibitors of predominantly nuclear HDACs. Saha was the first HDACi approved for clinical use in cancer therapy (advanced cutaneous T cell lymphoma) by the US Food and Drug Administration (FDA) [13]. Tsa is a potent antifungal antibiotic, isolated from a metabolite of *Streptomyces hygroscopicus* [14]. Vpa has been widely used in the treatment of epilepsy and as a mood stabilizer since the 1970s [15]. NaPB was approved by the US FDA for the treatment of hyperammonemia [16] and urea cycle disorders [17] and can be orally administrated in humans, safely achieving non-toxic millimolar plasma concentrations [18]. These four HDACis were selected in this work because they are already used in the clinic or are currently being evaluated in clinical trials of various diseases, manifesting a generally favorable toxicity profile [19–21]. This is the first study attempting to investigate the therapeutic effects of HDACis in combination with visible light-mediated PDT against cancer (we also refer the reader to the recent review covering previous and ongoing combination treatments with HDACis) [11]. Our results show that HDACis differentially potentiate the antitumor efficacy of HY-PDT in CRC cells, overcoming their resistance to this drug and epigenetically reactivating the expression of *CDKN1A*, which was otherwise silenced in these cells.

## Results

### HDACis sensitize colorectal cancer cells to HY-PDT, with differential effects between hydroxamic acids and short-chain fatty acids

**Fig. 1:**
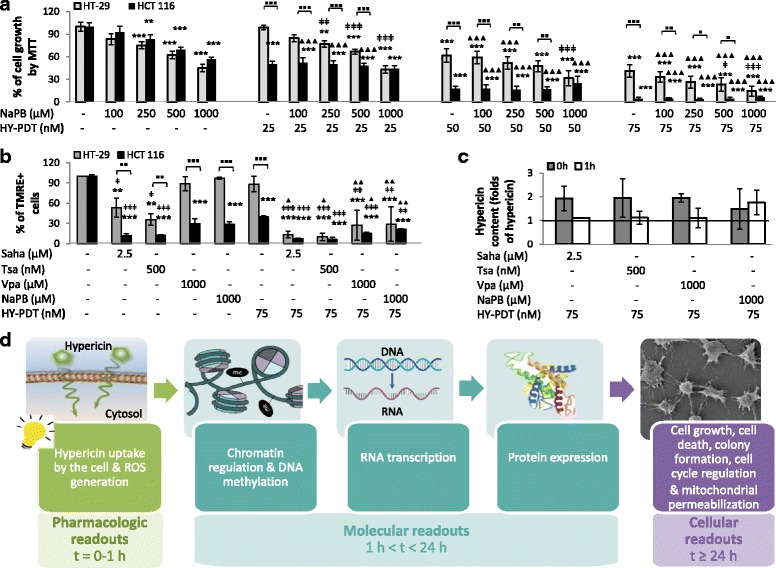
Effect of HDACis ± HY-PDT on cell growth and mitochondrial membrane dissipation potential. **a** Cell growth (metabolic activity by MTT), **b** mitochondrial membrane potential (TMRE+), and **c** HY content were measured after a sequential treatment starting with HDACis for 24 h followed by activation with hypericin for 48 h (**a**, **b**; HT-29 and HCT 116 cells) or for 0–1 h (**c**; HT-29 cells). The 0 h time point indicates that measurements were done immediately after hypericin activation. Samples treated with drug-free vehicle solvents (<0.1% DMSO) were used as the reference control. The results are expressed as percentage of control and represent the average ± SD of three independent experiments each done in triplicates (**a**) or singlets (**b**, **c**). Data was analyzed using one-way ANOVA with Tukey post hoc test. HCT 116 cell growth was compared to that of HT-29 (▪*p* < 0.05, ▪▪*p* < 0.01, ▪▪▪*p* < 0.001; **a**, **b**). All conditions were compared to the reference control (*p < 0.05, **p < 0.01, ***p < 0.001), and the combined treatments were compared to HY-PDT alone (ǂ*p* < 0.05, ǂǂ*p* < 0.01, ǂǂǂ*p* < 0.001) and to correspondingly equal concentrations of HDACis alone (▲*p* < 0.05, ▲▲*p* < 0.01, ▲▲▲*p* < 0.001). The study design is outlined in **d**

CRC cells are known to be resistant to HY-PDT [10]. We tested the antitumor effect of HY-PDT on two colon adenocarcinoma cell lines, HCT 116 and HT-29, and found that HT-29 cells were more resistant than HCT 116 at all tested HY-PDT concentrations (Fig. 1a, b). For example, 25 nM HY-PDT did not significantly affect the growth of HT-29 cells but decreased the proliferation of HCT 116 by 50% (Fig. 1a). Similarly, 75 nM HY-PDT did not significantly alter the mitochondrial membrane potential (measured as the percentage of TMRE+ stain) in HT-29 cells but caused 60% mitochondrial membrane dissipation (indicative of cell death) in HCT 116 cells (Fig. 1b). Therefore, we tested whether the HT-29 resistance to HY-PDT could be attenuated by pretreatment of cells with HDACis (this protocol is referred to herein as a “combination treatment”). Combination treatment of HY-PDT with the short-chain fatty acid HDACi NaPB markedly reduced the proliferation of HT-29 cells in a dose-dependent response to either drug in the combination (Fig. 1a). Unlike HY-PDT single treatment, NaPB, alone, did not differentially affect the cell growth of HT-29 versus HCT 116 cells at any tested concentration (Fig. 1a), showing that the two cell lines are equally sensitive to NaPB (*p* > 0.05), though not to HY-PDT (*p* < 0.05). The differential sensitivity to HY-PDT, however, was no longer observed between the two cell lines at any tested concentration of HY-PDT when the cells were pretreated with 1000 μM NaPB (Fig. 1a). In addition to causing similar growth inhibitory effects in both cell types, the combination treatment reduced the proliferation of both cell lines to less than 15% at 75 nM and 1000 μM concentrations of HY-PDT and NaPB, respectively, hence, overcoming the observed and known resistance of HT-29 cells to HY-PDT (Fig. 1a). The combination at these drug concentrations also caused mitochondrial membrane dissipation effects that are similar between both cell lines, reaching at least 71% dissipation in either cell type (Fig. 1b).

These results were encouraging to expand the set of tested HDACis to include another HDACi, Vpa, from the same chemical family as NaPB (short-chain fatty acid) and two other HDACis, Saha and Tsa, which belong to the chemical group of hydroxamic acids. Similarly to the NaPB concentrations tested above (NaPB 500 and 1000 μM), we selected HDACi inhibitory concentrations (IC) that were non-cytotoxic to HT-29 cells (IC < 50: Saha 1 and 2.5 μM, Tsa 250 and 500 nM, and Vpa 500 and 1000 μM) after 24 h (data not shown). At IC < 50 concentrations, the hydroxamic acids caused greater decreases in mitochondrial membrane potential than the short-chain fatty acids in both cell lines (Fig. 1b and Additional file 1: Figure S1). However, in combination with HY-PDT, both HDACi groups induced similar and high levels of mitochondrial membrane dissipation (Fig. 1b). This also indicated that the combination treatment renders HT-29 and HCT 116 cells similarly sensitive to each other and alters the mitochondrial membrane potential more effectively than single treatments. Moreover, differential antitumor properties of the drug combinations were not due to differential induction of HY intracellular accumulation (Fig. 1c). Because both cell lines exhibited similar sensitivities to the combination treatments, subsequent analyses focused on the HT-29 cell line, which was the model more resistant to single-drug treatments.

This and further analyses are structured throughout the manuscript according to the experimental design outlined in Fig. 1d. Specifically, three major readouts are analyzed in sequential order of mechanisms and time points: (1) pharmacologic, (2) molecular, and (3) cellular readouts. Pharmacologic readouts, such as drug uptake, are early events and, hence, measured at early time points. They subsequently initiate signaling events that can be measured as molecular readouts, and the latter eventually lead to changes in cell fate, such as cell growth or death (cellular readouts) (Fig. 1d). Moreover, the molecular readouts are more likely to be causal if they are detected at time points earlier than those at which the cellular readouts are observed; this is because, otherwise, the molecular events can more likely be a result (rather than a cause) of the changes in cell fates.

**Fig. 2:**
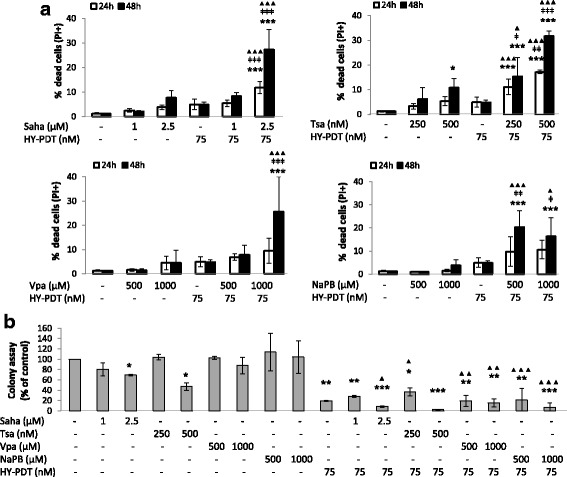
HT-29 cell death and colony formation potential by HDACis ± HY-PDT. **a** Cell death (PI+) was measured after a sequential treatment starting with the HDACis Saha, Tsa, Vpa, and NaPB (for 24 h) followed by activation with hypericin (for 24 or 48 h, as indicated). **b** Colony formation ability was measured after a sequential treatment starting with HDACis (for 24 h) followed by activation with hypericin (for 48 h) and subsequent seeding in tissue culture plates (for 10 days). Samples treated with drug-free vehicle solvents (<0.1% DMSO) were used as the reference control. The results are expressed as percentage of control and represent the average ± SD of **a** four and **b** three independent experiments each done in singlets. Data was analyzed using one-way ANOVA with Tukey post hoc test. All conditions were compared to the reference control (*p < 0.05, **p < 0.01, ***p < 0.001), and the combined treatments were compared to HY-PDT alone (ǂ*p* < 0.05, ǂǂ*p* < 0.01, ǂǂǂ*p* < 0.001) and to correspondingly equal concentrations of HDACis alone (▲*p* < 0.05, ▲▲*p* < 0.01, ▲▲▲*p* < 0.001)

Following the experimental design, HT-29 cell death was then analyzed by propidium iodide (PI)-based flow cytometry after cell treatment with each of the four HDACis followed by HY-PDT (Fig. 2a). None of the single treatments caused greater than 11% cell death at 24–48 h; however, HY-PDT (75 nM), in combination with HDACi IC < 50 concentrations, increased cell death proportions, with similar effects observed between hydroxamic and short-chain fatty acids (Fig. 2a). Similarly, HT-29 colony formation potential significantly decreased in the combination treatments relative to the single drugs, and, with the exception of 500 nM Tsa, the single treatments had modest effects on colony formation potential (Fig. 2b). For subsequent analyses, only concentrations of 2.5 μM Saha, 500 nM Tsa, 1000 μM Vpa, and 1000 μM NaPB were used in the combination treatments because these doses were more effective than lower concentrations in inducing cell death (Fig. 2a) or reducing colony formation (Fig. 2b). Altogether, these results converge to a common observation highlighting that HDACis sensitize CRC cells to the antitumor effect of HY-PDT. In single treatments, the hydroxamic acids decreased mitochondrial membrane potential and colony formation more strongly than the short-chain fatty acids at IC < 50 values. However, in combination with HY-PDT, both groups showed similar potencies against CRC cells.

### HDACi and HY-PDT combination treatments differentially modulate *HDAC* and *CDKN1A* expression, histone acetylation, and cell cycle regulation

**Fig. 3:**
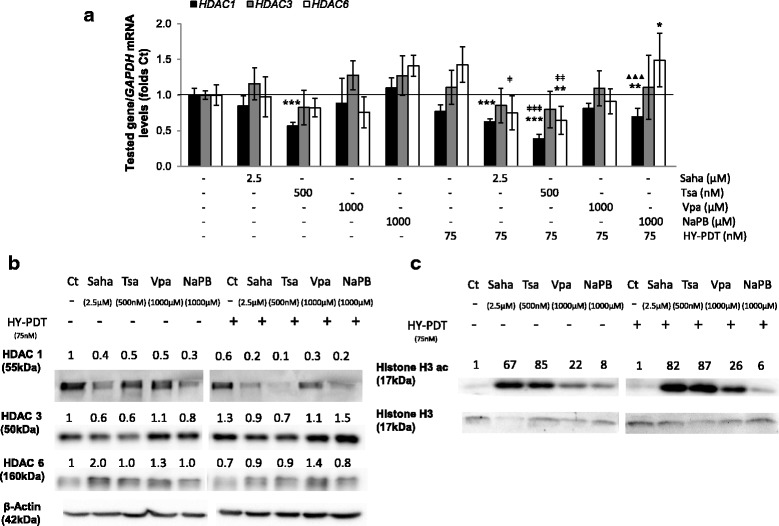
Effect of HDACis ± HY-PDT on HDAC expression and histone acetylation. **a** mRNA (by qRT-PCR) and **b**, **c** protein levels (by Western blotting) were measured in HT-29 cells after a sequential treatment starting with HDACis (for 24 h) followed by activation with hypericin for 8 h (**a**–**c**). Samples treated with drug-free vehicle solvents (<0.1% DMSO) were used as the reference control (Ct). Values are expressed as fold of Ct and represent the average ± SD of three independent experiments each done in triplicates. *HDAC* mRNA levels were normalized relative to those of the housekeeping gene, *GAPDH*. **b** HDAC and **c** histone H3 acetylation protein levels were quantified by densitometry, normalized relative to the loading controls (beta-actin and histone H3, respectively), and expressed as folds of the reference Ct. Western blots are representative of two independent experiments. Data was analyzed using one-way ANOVA with Tukey post hoc test. All conditions were compared to the reference Ct (*p < 0.05, **p < 0.01, ***p < 0.001), and the combined treatments were compared to HY-PDT alone (ǂ*p* < 0.05, ǂǂ*p* < 0.01, ǂǂǂ*p* < 0.001) and to correspondingly equal concentrations of HDACis alone (▲*p* < 0.05, ▲▲*p* < 0.01, ▲▲▲*p* < 0.001)

HDACis, in combination with HY-PDT (for 8 h), reduced the messenger RNA (mRNA) expression of *HDAC* genes (*HDACs 1*, *3*, and *6*) more strongly than either corresponding treatment alone (Fig. 3a). Moreover, in combination with HY-PDT, the hydroxamic acids reduced the expression of all three tested *HDACs* (though only *HDACs 1* and *6* being statistically significant) while the short-chain fatty acids reduced the expression of only *HDAC1* (with only NaPB effects being statistically significant) at IC < 50 values (Fig. 3a). Similar effects were observed on HDAC protein regulation at the same time point, wherein, in combination with HY-PDT, the hydroxamic acids reduced the protein levels of all three HDACs (though more modestly for HDACs 3 and 6 than HDAC1) while the short-chain fatty acids decreased mostly HDAC1 protein levels (Fig. 3b). Within the combination treatments, the stronger decrease in *HDAC* expression at 8 h by the hydroxamic acids was concomitant with a stronger induction of histone H3 acetylation at the same time point, compared to the short-chain fatty acids (Fig. 3c). Histone acetylation increased by 82–87 versus 6–26 folds relative to drug-free control when the combination treatments included hydroxamic acids versus short-chain fatty acids, respectively (Fig. 3c).

**Fig. 4:**
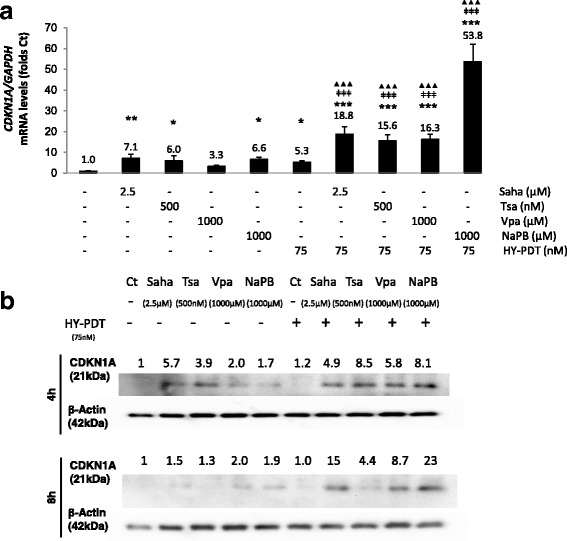
Effect of HDACis ± HY-PDT on *CDKN1A* expression. **a** mRNA (by qRT-PCR) and **b** protein levels (by Western blotting) levels were measured in HT-29 cells after a sequential treatment starting with HDACis (for 24 h) followed by activation with hypericin for 8 h (**a**, **b**) or 4 h (**b**), as indicated. Samples treated with drug-free vehicle solvents (<0.1% DMSO) were used as the reference control (Ct). Values are expressed as fold of Ct and represent the average ± SD of three independent experiments each done in triplicates. *CDKN1A* mRNA levels were normalized to relative to those of the housekeeping gene, *GAPDH*. **b** CDKN1A protein levels were quantified by densitometry, normalized relative to the loading control (beta-actin), and expressed as folds of the reference Ct. Ct protein levels were undetectable, so their values were set as 1.0 for reference purposes. Western blots are representative of two independent experiments. Data was analyzed using one-way ANOVA with Tukey post hoc test. All conditions were compared to the reference Ct (*p < 0.05, **p < 0.01, ***p < 0.001), and the combined treatments were compared to HY-PDT alone (ǂ*p* < 0.05, ǂǂ*p* < 0.01, ǂǂǂ*p* < 0.001) and to correspondingly equal concentrations of HDACis alone (▲*p* < 0.05, ▲▲*p* < 0.01, ▲▲▲*p* < 0.001)

**Fig. 5:**
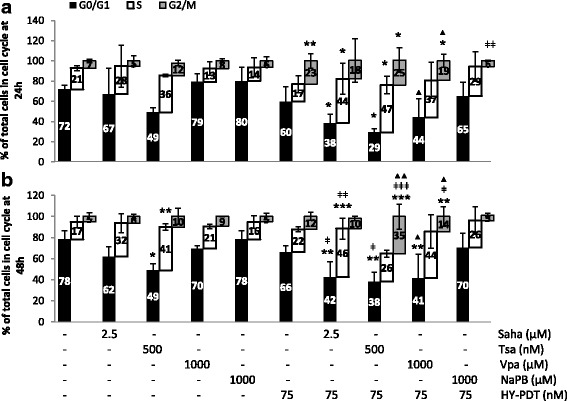
Effect of HDACis ± HY-PDT on cell cycle regulation. HT-29 cell cycle profiles (by PI-based flow cytometry) were measured after a sequential treatment starting with HDACis (for 24 h) followed by activation with hypericin for 24 h (**a**) or 48 h (**b**), as indicated. Samples treated with drug-free vehicle solvents (<0.1% DMSO) were used as the reference control. The results are expressed as percentage of total cycling cells and represent the average ± SD of three independent experiments each done in singlets. Data was analyzed using one-way ANOVA with Tukey post hoc test. All conditions were compared to the reference control (*p < 0.05, **p < 0.01, ***p < 0.001), and the combined treatments were compared to HY-PDT alone (ǂ*p* < 0.05, ǂǂ*p* < 0.01, ǂǂǂ*p* < 0.001) and to correspondingly equal concentrations of HDACis alone (▲*p* < 0.05, ▲▲*p* < 0.01, ▲▲▲*p* < 0.001)

Among the genes whose expression is highly coordinated by HDACi-mediated chromatin modulation is *CDKN1A* [8, 9]. Concomitant with the increased H3 acetylation (Fig. 3c), *CDKN1A* mRNA and protein expression were increased (Fig. 4a, b) by the combination treatments based on either the hydroxamic or the short-chain fatty acids. In particular, the combination treatments caused stronger increases in *CDKN1A* mRNA levels (by at least 15 folds relative to control) than either treatment alone (3.3–7.1-fold increases relative to control) (Fig. 4a). Similar trends were observed on CDKN1A protein regulation (Fig. 4b). Interestingly, CDKN1A protein levels were below the detection limits in drug-free controls and were “induced” (rather than just increased) by HDACi single or combination treatments (Fig. 4b). CDKN1A inhibits cyclin-dependent kinases that play a direct role in G1–S transition [22], and *CDKN1A* overexpression can cause S-phase arrest [23]. In fact, among the single treatments, the hydroxamic acids caused earlier (by 4 h rather than 8 h) and greater increases in CDKN1A protein levels (Fig. 4b), concomitant with larger increases in the proportion of S-phase-arrested cells, relative to the short-chain fatty acids (Fig. 5a, b). However, when used in combination treatments, both drug groups increased CDKN1A protein levels as early as 4 h (by 4.9–8.5 folds relative to control) and increased at 24 h the proportions of S-phase-arrested cells relative to control and to corresponding single treatments (Fig. 5a). Similar trends in cell cycle regulation were observed at 48 h (Fig. 5b). Overall, these results show that, in single or combination treatments, the hydroxamic acids show broader pan-inhibitory effects of HDACs and greater increases in global histone acetylation and in S-phase-arrested cells than the short-chain fatty acids at IC < 50 values. As expected, both groups of compounds induce *CDKN1A* expression.

### Sodium phenylbutyrate in combination with HY-PDT selectively modulates chromatin accessibility through histone acetylation at regulatory elements of the *CDKN1A* gene

In combination with HY-PDT, both HDACi chemical groups had similar potentials to inhibit colony formation and induce mitochondrial membrane dissipation in HT-29 cells (Fig. 1b, c). However, the hydroxamic acids were less selective than the short-chain fatty acids in their inhibitory potential towards HDACs versus other key epigenetic enzymes (Additional file 2: Figure S2.A). The former, in single or combination treatments with HY-PDT, not only inhibited HDAC expression (Fig. 3a, b) but also caused stronger decreases in mRNA levels of DNA and histone methyltransferases than the latter (Additional file 2: Figure S2.A). A particular observation was the fact that Vpa and NaPB increased, rather than inhibited, the expression of *DNMT3A*, but this effect was significantly attenuated when these HDACis were combined with HY-PDT (Additional file 2: Figure S2.A). Given that, in combination treatments, the short-chain fatty acids and hydroxamic acids had shown similar growth-inhibitory properties against HT-29 cells, the increased selectivity of the former towards inhibition of HDACs but not other key epigenetic enzymes made them a more interesting group for further investigation aiming to study the effect of HDACis on chromatin regulation by histone acetylation.

**Fig. 6:**
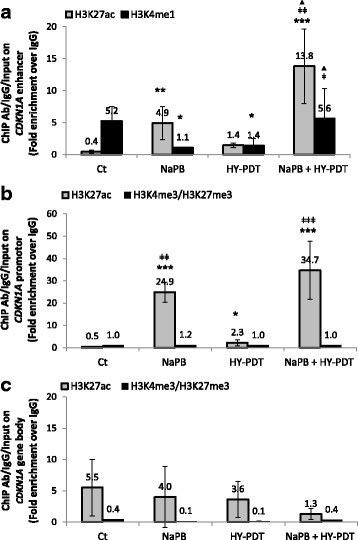
HDACi ± HY-PDT differentially modulates chromatin structure in the vicinity of *CDKN1A* regulatory regions. HT-29 cells were treated 24 h with NaPB (1000 μM) followed by 8 h of activation with hypericin (75 nM). Samples treated with drug-free vehicle solvents (<0.1% DMSO) were used as the reference control (Ct). **a** Enhancer, **b** promoter, and **c** gene body regulatory regions in the *CDKN1A* gene were analyzed by qPCR coupled to chromatin immunoprecipitation (ChIP) of histone modifications that mark each region element. Threshold cycle (Cq) values of Ct qPCR samples were similar. ChIP data was normalized over input DNA and IgG loading control and presented as the average ± SD of two independent experiments each done in triplicates. Data was analyzed using one-way ANOVA with Tukey post hoc test. All conditions were compared to the reference Ct (*p < 0.05, **p < 0.01, ***p < 0.001), and the combined treatments were compared to HY-PDT alone (ǂ*p* < 0.05, ǂǂ < 0.01, ǂǂǂ*p* < 0.001) and to equal concentrations of HDACis alone (▲*p* < 0.05, ▲▲*p* < 0.01, ▲▲▲*p* < 0.001). *H3K27ac* histone 3 lysine 27 acetylated, *H3K4me1* and *H3K4me3* histone 3 lysine 4 mono- and trimethylated, respectively, *H3K27me3* histone 3 lysine 27 trimethylated

Among the short-chain fatty acids (as well as all tested HDACis), NaPB caused the strongest induction of *CDKN1A* mRNA and protein levels, in combination with HY-PDT (Fig. 4). Hence, NaPB was chosen to determine whether its capacity to upregulate *CDKN1A* expression associates with its potential ability to modulate chromatin accessibility through histone acetylation at regulatory regions of the *CDKN1A* gene. Histone acetylation decondenses chromatin, allowing the binding of transcriptional activators. In particular, acetylation of lysine 27 of histone 3 (H3K27ac) is a well-established marker of transcriptionally active chromatin regions that are in the vicinity of promoter or enhancer elements [24]. NaPB + HY-PDT significantly increased, relative to the control or HY-PDT, H3K27ac occupancy at both the enhancer and promoter regions of *CDKN1A* (Fig. 6a, b). Moreover, NaPB + HY-PDT caused larger increases than NaPB alone in the H3K27ac enrichment (Fig. 6a, b), concomitant with the stronger induction in *CDKN1A* expression by the former treatment (Fig. 4a, b). As H3K27ac can mark both enhancer and promoter regions, we also tested the enrichment of histone 3 lysine 4 monomethylation (H3K4me1), a marker specific to enhancer regions, whether active, poised, or primed [25]. Unlike in the single-drug treatment conditions, H3K4me1 was not decreased relative to control in the NaPB + HY-PDT (Fig. 6a), indicating that the transcriptionally active (marked by H3K27ac) chromatin regions localize to *CDKN1A* enhancer elements in the combination treatment. Given the observed property of NaPB, as a short-chain fatty acid, to specifically regulate the activity of HDACs but not other key epigenetic enzymes, we next tested whether this HDACi is able to specifically regulate histone acetylation, rather than other key histone modifications, at the *CDKN1A* gene. Histone 3 trimethylation at lysines 4 (H3K4me3) versus 27 (H3K27me3) mark transcriptionally active versus inactive regions, respectively. As both histone marks localize to promoter regions and represent modifications of the same histone, their ratio is used as a measure of chromatin accessibility [24]; the higher the ratio of H3K4me3/H3K27me3 in a given region, the more active is the chromatin in that region. NaPB, in single or combination treatments, did not modulate this ratio on the *CDKN1A* promoter relative to other treatment groups (Fig. 6b), concomitant with the unaltered expression of the key H3K27 histone methyltransferase, EZH2, observed after NaPB + HY-PDT treatments (Additional file 2: Figure S2A). To confirm the specificity towards enhancer and promoter elements of both the tested histone marks and the NaPB + HY-PDT-mediated chromatin decondensation, we analyzed the enrichment of H3K27ac, H3K4me3, and H3K27me3 on gene body regions of *CDKN1A* (Fig. 6c). As expected, there was no statistical difference in the enrichment of any of those histone marks on the *CDKN1A* gene body after treatments with NaPB ± HY-PDT, HY-PDT, or drug-free control (Fig. 6c).

**Fig. 7:**
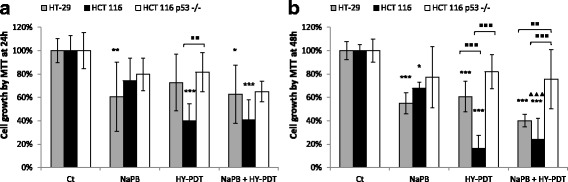
Effect of NaPB ± HY-PDT on the cell growth of HT-29, HCT 116, and HCT 116 p53−/− cells. Cell growth (metabolic activity by MTT) was measured after a sequential treatment starting with NaPB (1000 μM) for 24 h followed by activation with hypericin (75 nM) for 24 h (**a**) and 48 h (**b**). Samples treated with drug-free vehicle solvents (<0.1% DMSO) were used as the reference control. The results are expressed as percentage of control and represent the average ± SD of two independent experiments each done in quadruplicates. Data was analyzed using one-way ANOVA with Tukey post hoc test. Different cell types were compared to each other (▪*p* < 0.05, ▪▪*p* < 0.01, ▪▪▪*p* < 0.001; **a**, **b**), all conditions were compared to the reference control (*p < 0.05, **p < 0.01, ***p < 0.001), and the combined treatments were compared to correspondingly equal concentrations of HDACis alone (▲*p* < 0.05, ▲▲*p* < 0.01, ▲▲▲*p* < 0.001)

Furthermore, in comparison with HY-PDT or control, NaPB + HY-PDT did not affect DNA methylation levels of the *CDKN1A* gene enhancer (Additional file 3: Figure S3A), promoter (Additional file 3: Figure S3B), and gene body (Additional file 3: Figure S3C) regions, further supporting the observation that the epigenetic regulation of *CDKN1A* by NaPB + HY-PDT is through histone acetylation at enhancer and promoter elements rather than through histone or DNA methylation at those or other regulatory regions. These epigenetic and antitumor activities of NaPB were also not likely due to potentially indirect mechanisms of this drug to cause (1) higher intracellular accumulation, hence more efficient activation of PDT (Fig. 1c), or (2) increased generation of reactive oxygen species (Additional file 2: Figure S2.B) and associated DNA damage (Additional file 2: Figure S2.C). Moreover, because HT-29 has a gain-of-function R273H mutation in *P53* relative to the wild-type HCT 116 and given that *CDKN1A* is regulated by *P53*, we showed that the NaPB-mediated mechanism is not *P53*-dependent (Fig. 7). In particular, no significant differential sensitivity to NaPB was observed by comparing HCT 116 cells with their clonal *P53* null derivatives, HCT 116 p53−/− (or compared with HT-29 cells re-analyzed in the same experiment) (Fig. 7). However, a strong *P53*-dependent mechanism of HY-PDT was observed in the CRC cells (Fig. 7), as reported earlier by our group [26]. As for the NaPB + HY-PDT combination treatment, its growth inhibitory effects on both the HCT 116 and HCT 116 p53−/− cells were very similar to those of HY-PDT alone and different from those of NaPB single treatment. Hence, we conclude that the *P53* dependency of the combination treatment is likely driven by the HY-PDT rather than the NaPB constituent.

## Discussion

HY is a major biologically active and photosensitizing constituent of St. John’s wort [1], which is traditionally used in herbal infusions as a natural medicine; hence, HY could be suitable for chemoprevention strategies particularly that it is non-genotoxic. Photosensitizing agents used in cancer therapy, once injected into the bloodstream, are absorbed by cells all over the body but stay longer in cancer than in normal cells. Approximately 24 to 72 h after injection, when most of the photosensitizer has left normal but not tumor cells [27], the target tissue is exposed to light. The photosensitizer in the tumor absorbs the light and often produces reactive oxygen species that kill nearby cancer cells. Besides its high tumor selectivity, PDT has other advantages such as very good cosmetic outcomes with negligible scarring [28] as well as a broad range of total light and drug doses that allow multiple applications of PDT towards the same tumor (unlike radiation). To date, the FDA has approved photosensitizing agents for PDT-mediated cancer therapy of esophageal and non-small cell lung cancer, and clinical trials are undergoing for cancers of the brain, skin, prostate, cervix, and peritoneal cavity, including the intestines, stomach, and liver [29] (National Cancer Institute, USA).

Preclinical research on photosensitizers for CRC treatment has captured increased attention in 2016 [30], particularly that this cancer represents the third most common cancer worldwide in men and the second in women (10.0 and 9.2% of the total incidences, respectively) [31]. Human CRC cell line models have recently demonstrated increased resemblance to the corresponding tumor biology in patients as well as important pharmacological utility in preclinical research (including photosensitizer drugs) [32, 33]. For example, recent mutational and gene expression analyses of 151 CRC cell lines showed that the whole spectrum of CRC molecular and transcriptional subtypes, previously defined in patients, is represented in this cell line compendium [32]. Moreover, the human CRC lines are representative of the main subtypes of primary tumors at the genomic level, further validating their utility as tools to investigate CRC biology and drug responses [33]. Among the CRC cell lines, HT-29 and HCT 116 have been the most widely investigated models in PDT research [30], and HT-29 is known to be resistant to HY-PDT [10].

We have tested the effect of HY-PDT on both cell lines and observed that HT-29 cells were more resistant than HCT 116 at all tested HY-PDT concentrations (Fig. 1). However, pretreatment of the cells with HDACis rendered both cell lines equally and highly sensitive to HY-PDT (Fig. 1a: 1000 μM NaPB, and Fig. 1b). In addition, this combination treatment rendered the short-chain fatty acids similarly potent to the hydroxamic acids against HT-29 cells (Figs. 1 and 2). Otherwise, in the absence of HY-PDT, the hydroxamic acids were more potent (decreased colony growth and mitochondrial membrane potential) against HT-29 cells and were active at lower concentration ranges (0.5–2.5 μM), relative to the short-chain fatty acids, which had no effects up to 1000 μM concentrations (Figs. 1b and 2b). In the absence of HY-PDT, the two HDACi groups also exhibited differential activities at the mechanistic level. Specifically, the hydroxamic acids, compared to the short-chain fatty acids at IC < 50 values, had stronger and broader inhibitory potentials against different HDACs (Fig. 3b) and DNA and histone methyltransferases (Additional file 2: Figure S2.A), caused higher increases in histone acetylation (Fig. 3c), induced greater proportions of S-phase-arrested cells (Fig. 5), and stimulated stronger and earlier protein expression of *CDKN1A* (Fig. 4b). In comparison with both HDACi groups, HY-PDT, alone, had minor or no effects on these mechanistic parameters.

Given that, in combination treatments, the hydroxamic acids and short-chain fatty acids had shown similar growth-inhibitory properties against HT-29 cells, the increased selectivity of the latter towards inhibition of HDACs versus other key epigenetic enzymes (Additional file 2: Figure S2.A) made them more interesting candidates for studying chromatin regulation through histone acetylation. Moreover, in combination treatment, the short-chain fatty acids were able to upregulate CDKN1A protein expression as early as the hydroxamic acids (Fig. 4b). In particular, NaPB, when combined with HY-PDT, caused the strongest induction of *CDKN1A* expression among all tested HDACis (Fig. 4); therefore, we focused our analysis on NaPB to determine whether its ability to upregulate *CDKN1A* expression correlates with its potential to modulate chromatin accessibility through histone acetylation at regulatory regions of the *CDKN1A* gene. NaPB + HY-PDT significantly increased H3 acetylation at the *CDKN1A* gene (Fig. 6a, b), with less effects on global histone acetylation (also when compared to the other HDACis) (Fig. 3c), suggesting a more specific effect on the *CDKN1A* gene relative to global chromatin structure. In particular, the epigenetic regulation of *CDKN1A* by NaPB ± HY-PDT was associated to histone acetylation at enhancer and promoter elements rather than histone or DNA methylation at those or other regulatory regions. Notably, NaPB + HY-PDT caused the lowest levels of dead (≤20%; Fig. 2a) and arrested cells (Fig. 5) compared to all other HDACi + HY-PDT combination treatments. This ability to elicit selective epigenetic activities at concentrations that are not cytotoxic represents an important property of epigenetic drugs, which, at pharmacologically active concentrations, allow the cells to continue proliferation but exploit cell division for the purpose of amplifying or maintaining the epigenetic effect across cell generations [34].

Interestingly, the epigenetic effect of NaPB was not dependent on *P53* while the growth inhibitory potential of HY-PDT (which had minor or no epigenetic effects) was largely *P53*-dependent. We have previously investigated in more detail the *P53*-dependent mechanism of HY-PDT and observed a higher level of apoptosis in HCT 116 *P53* wild type than in *P53*−/− cells, with the *P53* null status causing resistance at later stages of programmed cell death [26]. The NaPB + HY-PDT combination treatment showed similar *P53*-dependent growth inhibition as HY-PDT alone on both the HCT 116 *P53* wild type and *P53*−/− cells, with different effects from the NaPB single treatment; hence, the *P53* dependency of the combination treatment is likely driven by the HY-PDT rather than the NaPB constituent.

## Conclusions

Our results show that HDACis potentiate the antitumor efficacy of HY-PDT in CRC cells, overcoming their resistance to this drug and epigenetically reactivating the expression of *CDKN1A*, which was otherwise silenced in these cells. This is the first study in solid or liquid cancers highlighting the efficacy of therapeutic regimens involving HDACis in combination with visible light-mediated PDT (including hypericin). One study recently reported that HDACis potentiate UVA-mediated phototherapy of T cell lymphoma [35], but UVA itself is known to be a risk factor for cancer [36]. The mechanism of coupling HDAC inhibition to PDT seems to have recently captured the interest of synthetic chemistry wherein two independent studies have each synthesized one new compound possessing both photoactivation and HDAC inhibitory potentials [37, 38]. Combination of PDT with HDAC inhibition represents a novel approach, with potentially promising outcomes, in cancer therapy. The fact that HDACis and HY also represent common and non-genotoxic constituents of dietary agents [1, 34, 39] makes them interesting targets for studies aiming to investigate mechanisms for dietary-based cancer prevention.

## Methods

### Aim, design, and setting of the study

Preclinical research on photosensitizers has captured increased attention very recently, particularly for CRC treatment, as this cancer is known to be resistant to PDT [30]. In this study, we attempted to overcome CRC cell resistance to HY-PDT by pretreatment with HDACis especially that the latter are non-genotoxic agents and can epigenetically sensitize cells to external stimuli. The mechanism of coupling HDAC inhibition to PDT seems to have recently captured the interest of synthetic chemistry wherein two independent studies have each synthesized one new compound possessing both photoactivation and HDAC inhibitory potentials [37, 38]. We tested two chemical groups of HDACis: (a) the hydroxamic acids, which are inhibitors of all classes of HDACs, and (b) the short-chain fatty acids, which are inhibitors of predominantly nuclear HDACs. Moreover, specific HDACis were tested in each group based on the criterion that they be in clinical use or trials and that they manifest a generally favorable toxicity profile [19–21]. The target CRC cell models selected were HCT 116, HCT 116 p53−/−, and HT-29 because they are the most widely investigated cell lines in PDT research [30], and HT-29 is known to be resistant to HY-PDT [10]. We hypothesized that chromatin regulation by HDACis, particularly at the *CDKN1A* tumor suppressor gene, could sensitize cancer cells to photochemical and photobiological processes induced by HY-PDT. This is the first study in solid or liquid cancers highlighting the efficacy of therapeutic regimens involving HDACis in combination with visible light-mediated PDT (including hypericin). The fact that HDACis and HY also represent common and non-genotoxic constituents of dietary agents [1, 34, 39] makes them interesting targets for studies aiming to investigate mechanisms for dietary-based cancer prevention.

### Cell culture

HT-29 and HCT 116 were obtained from the American Type Culture Collection (Rockville, MD, USA). HCT 116 p53−/− was a gift from Professor Bert Vogelstein (kindly provided by Dr. Alois Kozubık, Institute of Biophysics, Brno, Czech Republic). Cells were cultured in RPMI 1640 medium (Gibco, Grand Island, NY, USA) supplemented with 10% heat-inactivated fetal calf serum (FCS, PAA Laboratories GmbH, Austria) and 7.5% NaHCO_3_ (10 ml/l), penicillin 100 U/ml, streptomycin 100 mg/ml, and amphotericin 25 mg/ml (Invitrogen, Carlsbad, CA, USA) at 37 °C, 95% humidity, and 5% CO_2_.

### Treatment reagents and conditions

Hypericin (4,5,7,4,5,7-hexahydroxy-2,2-dimethylnaphtodiantron, AppliChem GmbH, Darmstadt, Germany), SAHA (Sigma-Aldrich, St. Louis, MO, USA), TSA (Sigma-Aldrich), VPA (Santa Cruz Biotechnology, Santa Cruz, CA, USA), and NaPB (Santa Cruz Biotechnology) were prepared in DMSO for stock solutions and then diluted to working concentrations. The final concentration of DMSO was less than 0.1%. HT-29 and HCT 116 cells were seeded in appropriate plates (96 wells, 6 wells, 60 mm or 100 mm) for 24 h and then treated at 50% confluency with HDACis ± HY-PDT according to the treatment sequence and time points specified in the “Results.” Briefly, cells were pretreated with a given HDACi or drug-free solvent control for 8 h, and then HY was added in the dark for 16 h, which is the optimal incubation time for HY intracellular accumulation. Subsequently, cells were exposed to light to activate hypericin and were cultured for further indicated time points. Light activation was performed using at a total dose of 3.15 J/cm^2^ (fluence rate 3.15 mW/cm^2^), which covers HY maximum absorbance (590–600 nm). The irradiation device consisted of 11 white L18W/30 lamps (Osram, Berlin, Germany) with a maximum emission range of 530–620 nm.

### MTT assay

HT-29 and HCT 116 cells were seeded in 96-well plates and treated at 50% confluency according to indicated treatment conditions. MTT (3-[4,5-dimethylthiazol-2-yl]-2,5-diphenyltetrazolium bromide) was added to cells 48 h after hypericin activation at a final concentration of 0.5 mg/ml. The reaction was stopped after 4 h of incubation at 37 °C by addition of sodium dodecyl sulfate (SDS) at a final concentration of 3.3% to dissolve insoluble formazan. The absorbance (*λ* = 584 nm) was measured using a BMG FLUOstar Optima (BMG Labtechnologies GmbH, Offenburg, Germany). Results were evaluated as percentages of the absorbance of the drug-free control.

### Mitochondrial membrane depolarization

HT-29 and HCT 116 cells were seeded in 60-mm dishes and treated at 50% confluency according to indicated treatment conditions, then harvested by trypsinization at 24 or 48 h after hypericin activation. The cells were collected together with floating cells (total 2 × 10^5^), washed with HBSS (Hank’s balanced salt solution), and stained with 0.1 μM tetramethylrhodamine ethyl ester perchlorate (TMRE; Sigma-Aldrich) in HBSS for 20 min at room temperature in the dark. Mitochondrial membrane potential was measured by flow cytometry (BD FACSCalibur, BD Biosciences, San Jose, CA, USA) and FlowJo software (TreeStar Inc., Ashland, OR, USA).

### Propidium iodide-based cell death assay

HT-29 cells were seeded in 60-mm dishes and treated at 50% confluency according to indicated treatment conditions, then harvested by trypsinization at 24 or 48 h after hypericin activation. The cells were collected together with floating cells (total 2 × 10^5^), washed with HBSS, and stained with 25 μg/ml propidium iodide (PI, Sigma-Aldrich) in HBSS. Cell death (PI+ cells) was measured by flow cytometry (BD FACSCalibur) and FlowJo software (TreeStar Inc.)

### Colony formation assay

HT-29 cells were seeded in 60-mm dishes and treated at 50% confluency according to indicated treatment conditions. Forty-eight hours after hypericin activation, cells were harvested by trypsinization, and 500 cells per well were seeded in 6-well plates and cultured for 10 days. The plates were then stained with methylene blue dye (0.8% w/v) and scanned. Colonies were counted using Clono-Counter software [40].

### RNA isolation, RT, and qRT-PCR

HT-29 cells were seeded in 60-mm dishes and treated at 50% confluency according to indicated treatment conditions, then harvested by trypsinization at 8 h after hypericin activation. Total RNA was then isolated using the TRIzol Reagent (Invitrogen) according to the manufacturer’s instructions and stored at −80 °C. RNA quantity and quality were assessed with a ND-8000 spectrophotometer (NanoDrop, Thermo Scientific, Wilmington, USA). Reverse transcription (RT) reactions were performed using MMLV-RT (Invitrogen) and random hexamers on 500 ng of total RNA per reaction according to the manufacturer’s protocol. Quantitative RT-PCR (qRT-PCR) was performed in triplicate for each sample. The genes tested were *HDAC1*, *HDAC3*, *HDAC6*, *CDKN1A*, *DNMT1*, *DNMT3A*, *EZH2*, and *GAPDH*, which was used as a reference gene. The PCR conditions used were as follows: 95 °C 5 min, [95 °C 15 s, 60 °C 30 s] × 40 cycles, 95 °C 1 min, and pause 4 °C. The primers used are reported in Table 1. The assays were performed using MESA GREEN qPCR MasterMix Plus (Eurogentec, Seraing, Belgium) and a CFX96 RealTime PCR Detection System (Bio-Rad Laboratories). mRNA levels were calculated using the 2−ΔCt method (ΔCt = ΔCt target gene − ΔCt reference gene).

**Table 1:** Primers used

Method	Gene	Primers (5′-to-3′ sequence)
qRT-PCR	*HDAC1*	fw CCAAGTACCACAGCGATGAC rev CTGGACAGTCCTCACCAACG
	*HDAC3*	fw TTGAGTTCTGCTCGCGTTACA rev CCCAGTTAATGGCAATATCACAGAT
	*HDAC6*	fw TGGCTATTGCATGTTCAACCA rev GTCGAAGGTGAACTGTGTTCCT
	*CDKN1A*	fw GACACCACTGGAGGGTGACT rev CCACATGGTCTTCCTCTGCT
	*DNMT1*	fw GATGTGGCGTCTGTGAGGT rev CCTTGCAGGCTTTACATTTCC
	*DNMT3A*	fw CCTGAAGCCTCAAGAGCAGT rev TGGTGTCCTTCTGTTCTTTGC
	*EZH2*	fw ACTGGCGAAGAGCTGTTTTT rev TTCGATGCCGACATACTTCA
	*GAPDH*	fw AACGGGAAGCTTGTCATCAA rev TGGACTCCACGACGTACTCA
Pyrosequencing	*CDKN1A* enhancer	fw AGGAGGGAAGTGTTTTTTTGTAGTA rev biotinylated-ACAACTACTCACACCTCAACTAAC sequencing primer: fw primer was used as sequencing primer in addition to: S1 GGGTAGTTAGGAGT S2 TTTGGTTTTTTTGAG Genomic coordinates (hg38) of spanned region: CHRO STRAND 6 +, START 36678496, END 36678730
	*CDKN1A* promoter	fw GGTTGGAATAGTTTGTTTTTAAGGA rev biotinylated-AAATAAAAAAAACCCTTACCCTTC sequencing primer: fw primer was used as sequencing primer in addition to: S GCGCGTTGTAGGG Genomic coordinates (hg38) of spanned region: CHRO STRAND 6 +, START 36679579, END 36679743
	*CDKN1A* gene body	fw GGGTTTTGGTTTGTTTAAGTTTTATT rev biotinylated-CCACATAATCTTCCTCTACTATCC sequencing primer: fw primer was used as sequencing primer Genomic coordinates (hg38) of spanned region: CHRO STRAND 6 +, START 36684308, END 36684436
ChIP	*CDKN1A* enhancer	fw GAAGCATGTGACAATCAACAACT rev AAGCATCTTGAGGCCAGAAT Genomic coordinates (hg38) of spanned region: CHRO STRAND 6 +, START 36678037, END 36678143
	*CDKN1A* promoter	fw ACTCCAGAAGCCCTCTCC rev GGCTTCCTTGGGAACAAACT Genomic coordinates (hg38) of spanned region: CHRO STRAND 6 +, START 36679513, END 36679607
	*CDKN1A* gene body	fw GAGACCCTCTGGTAGGAAGA rev GAGATACAAGGAAGGCCCTG Genomic coordinates (hg38) of spanned region: CHRO STRAND 6 +, START 36683986, END 36684088

### Western blot analysis

HT-29 cells were seeded in 100-mm dishes and treated at 50% confluency according to indicated treatment conditions, then harvested by trypsinization at 8 h after hypericin activation. Western blot analyses were conducted using total cellular protein extracts (30–50 mg). The blots were incubated overnight at +4 °C with the following specific primary antibodies: anti-HDAC1 (1/1000, ab 46985, Abcam), anti-HDAC3 (1/500, sc-11417, Santa Cruz Biotechnology), anti-HDAC6 (1/500, sc-11420, Santa Cruz Biotechnology), anti-CDKN1A (1/500, sc-397, Santa Cruz Biotechnology), anti-H3 (1:2500, ab 1791, Abcam), anti-H3ac (1:2500, MILL 17-245, Merck Millipore), followed by incubation with species-matched secondary antibodies. β-Actin (1:5000, A5441, Sigma-Aldrich) or H3 was used as the loading control. Specific proteins were detected by exposing membranes to ChemiDoc XRS+ System (Bio-Rad Laboratories) after incubation with Pierce ECL Western Blotting Substrate (Thermo Fisher Scientific). Densitometry analysis was performed using ImageJ software.

### Cell cycle analysis

HT-29 cells were seeded in 60-mm dishes and treated at 50% confluency according to indicated treatment conditions, then harvested by trypsinization at 24 or 48 h after hypericin activation, fixed in cold 70% ethanol, and kept at +4 °C overnight. Prior to analysis, cells were washed the next day twice in PBS, mixed with staining solution (0.1% Triton X-100, 0.137 mg/ml ribonuclease A, 20 μg/ml PI), and incubated in the dark for 30 min at room temperature. Cell cycle profiles (1.5 × 10^4^ cells per sample) were analyzed by flow cytometry (BD FACSCalibur), and ModFit 3.0 software (Verity Software House, Topsham, ME, USA) was used to generate DNA content frequency histograms and to quantify the percentage of cells in the individual cell cycle phases.

### ChIP assay

HT-29 cells were seeded in 100-mm dishes and treated at 50% confluency according to indicated treatment conditions, then harvested by trypsinization at 8 h after hypericin activation, washed in PBS, counted, and diluted to five million living cells in 500 μl of PBS for sampling. Chromatin proteins of interest were cross-linked to DNA by addition of formaldehyde to a final concentration of 1%, and the cells were incubated at 37 °C for 8 min and quenched with 0.125 M glycine for 5 min at room temperature. Lysis was done using Chromatin shearing kit - Low SDS (Diagenode, Seraing, Belgium) according to the manufacturer’s protocol. Lysates were sonicated to reduce the size of DNA to 200–500 bp as determined by agarose gel electrophoresis. Chromatin immunoprecipitation (ChIP) was then carried out on the Diagenode Automated Platforms SX-8G IP-Star® Compact using auto histone ChIP-seq kit (Diagenode) and the following antibodies: H3K4me1 (2.5 mg/ml, Ab 8895, Abcam), H3K27ac (2.5 mg/ml, Ab 4729, Abcam), H3K27me3 (2.5 mg/ml, Ab 6002, Abcam), H3K4me3(2.5 mg/ml, Ab1012-100, Abcam), IgG (0.2 ng/ml, C15410206, Diagenode). The enrichment of specific DNA regions in the immune-precipitated chromatin was measured by qPCR using primers spanning the *CDKN1A* enhancer, promoter, and gene body region. These regulatory elements were determined using the UCSC Genome Browser. The primers used are reported in Table 1. Amplification of the immunoprecipitated DNA was achieved using the MESA GREEN qPCR MasterMix Plus for SYBR Assay buffer (Eurogentec). The qPCR was performed with a CFX96 Touch Real-Time System (Bio-Rad Laboratories). The PCR conditions used were as follows: 95 °C 5 min, [95 °C 15 s, 60 °C 30 s] × 40 cycles, 95 °C 1 min, and pause 4 °C. Fold enrichment in each immunoprecipitation was determined by normalizing the intensities of the PCR product in immunoprecipitated DNA to the amount of input DNA (total chromatin before immunoprecipitation) and to IgG control. Only 10% of the total input was used in the PCR reactions. ChIP assays were repeated two times using different chromatin preparations.

### Intracellular accumulation of hypericin

HT-29 cells were seeded in 60-mm dishes and treated at 50% confluency according to indicated treatment conditions. Immediately or 1 h after HY activation, cells were harvested by trypsinization and collected together with floating cells (total 2 × 10^5^), washed in PBS, and resuspended in HBSS. HY intracellular content was measured by flow cytometry (BD FACSCalibur) and FlowJo software (TreeStar Inc.) and evaluated as the ratio of relative fluorescence of combined treatment compared to the relative fluorescence of each of HDACi and HY.

### Production of reactive oxygen species

HT-29 cells were seeded in 60-mm dishes and treated at 50% confluency according to indicated treatment conditions. Immediately or 1 h after HY activation, cells were harvested by trypsinization and collected together with floating cells (total 2 × 10^5^), then washed twice in PBS and resuspended in HBSS with dihydrorhodamine-123 (DHR-123, Fluka, Buchs, Switzerland) at a final concentration of 0.2 μM. The samples were then incubated for 15 min at 37 °C in 5% CO_2_, and total reactive oxygen species (ROS) was measured by flow cytometry (BD FACSCalibur) and FlowJo software (TreeStar Inc.).

### Measurement of histone H2AX phosphorylation

HT-29 cells were seeded in 60-mm dishes and treated at 50% confluency according to indicated treatment conditions. Hydrogen peroxide (2 mM H_2_O_2_) was used as positive control and was added to the cells for 30 min (22 h 30 min after hypericin activation), and afterwards, the medium was replaced with fresh medium for 1 h. Cells were then harvested by trypsinization at 24 h after hypericin activation, fixed, permeabilized, and washed using BD Cytofix/Cytoperm™ Fixation/Permeabilization Solution Kit (BD Biosciences), according to the manufacturer’s instructions. After 20 min of incubation in 3% FBS and centrifugation, cells were stained for 1 h at room temperature in the dark with antibodies against either IgG1 κ Isotype Control (1:10, BD Biosciences) or histone H2AX phosphorylation on Ser139 (γH2AX) (1:10, BD Biosciences), which is a marker of DNA double-strand breaks (DSBs). Staining intensity was quantified by flow cytometry (BD FACSCalibur) and FlowJo software (TreeStar Inc.). The expression of γH2AX was expressed as a ratio of the median fluorescence of anti-H2AX (pS139) to that of IgG1 κ Isotype Control.

### DNA methylation analysis: DNA extraction, bisulfite conversion, and pyrosequencing

HT-29 cells were seeded in 60-mm dishes and treated at 50% confluency according to indicated treatment conditions, then harvested by trypsinization at 8 h after hypericin activation, pelleted, resuspended in lysis buffer (1% SDS, 0.1 M NaCl, 0.1 M EDTA, 0.05 M Tris; pH 8) with proteinase K (500 μg/ml), and incubated for 2 h at 55 °C. Saturated NaCl (6 M) was added, DNA was precipitated with isopropanol, and cleaned with 70% ethanol. Extracted DNA was resuspended in water. Quantity and quality of the extracted DNA were assessed with a ND-8000 spectrophotometer (NanoDrop, Thermo Scientific). To quantify the percentage of methylated cytosine in individual CpG sites, we performed bisulfite pyrosequencing, as described [41]. Briefly, bisulfite conversion was performed on 500 ng of DNA using the EZ DNA Methylation Kit (Zymo Research) following the manufacturer’s recommendations. The efficacy of bisulfite modification was confirmed by PCR using primers specific for bisulfite-converted versus unconverted DNA in the *GAPDH* gene. The regions of interest (10 to 25 ng of converted DNA) were amplified by PCR and pyrosequenced (PSQ 96MA, Biotage) using PyroGold Reagent kit (Qiagen). The percentage of methylation for each CpG was calculated as the mean methylation of all CpGs analyzed at that genetic position. Primers for PCR, sequencing primers, and regions are described in Table 1.

### Statistical analyses

SPSS Version 16.0 and Microsoft Office Excel 2010 were used to perform the statistical measurements and comparisons. Data distributions showed conformity with assumptions of normality and equality of variances, and, accordingly, parametric tests were performed (independent sample *t* test and ANOVA with associated post hoc tests: Dunnett’s *t* and Tukey), as indicated in the figure legends. Statistical significance was claimed when the *p* value was ≤0.05.

## Additional files

Additional file 1: Figure S1. Mitochondrial membrane dissipation potential by HDACis ± HY-PDT. Mitochondrial membrane dissipation (TMRE+) was measured in HT-29 cells after a sequential treatment starting with HDACis (A) Saha, (B) Tsa, (C) Vpa, and (D) NaPB (for 24 h) followed by activation with hypericin (for 24 or 48 h, as indicated). Samples treated with drug-free vehicle solvents (<0.1% DMSO) were used as the reference control. The results are expressed as the percentage of control and represent the average ± SD of four independent experiments each done in singlets. Data was analyzed using one-way ANOVA with the Tukey post hoc test. All conditions were compared to the reference control (*p < 0.05, **p < 0.01, ***p < 0.001), and the combined treatments were compared to HY-PDT alone (ǂ*p* < 0.05, ǂǂ*p* < 0.01, ǂǂǂ*p* < 0.001) and to correspondingly equal concentrations of HDACis alone (▲*p* < 0.05, ▲▲*p* < 0.01, ▲▲▲*p* < 0.001) (PPTX 55 kb).

Additional file 2: Figure S2. Effect of HDACis ± HY-PDT on DNA and histone methyltransferase expression and DNA damage signaling. Measurements were performed in HT-29 cells after a sequential treatment starting with HDACis for 24 h followed by activation with hypericin for 8 h (A), 0–1 h (B), or 24 h (C), as indicated. The 0 h time point indicates that measurements were done immediately after hypericin activation. Samples treated with drug-free vehicle solvents (<0.1% DMSO) were used as the reference control (Ct). (A) mRNA (by qRT-PCR) levels are expressed as fold of Ct and represent the average ± SD of three independent experiments each done in triplicates. *DNMT1*, *DNMT3A*, and *EZH2* mRNA levels were normalized relative to those of the housekeeping gene, *GAPDH*. (B) Total reactive oxygen species (ROS) and (C) histone H_2_AX phosphorylation levels represent the average ± SD of three independent experiments each done in singlets. H_2_O_2_ (2 M) was used as a positive treatment control for DNA damage mediated by H_2_AX phosphorylation (C). Phosphorylated H_2_AX protein levels (by FACS) were normalized relative to those of isotype H_2_AX. Data was analyzed using one-way ANOVA with the Tukey post hoc test and Dunnett’s multiple comparison test. All conditions were compared to the reference Ct (*p < 0.05, **p < 0.01, ***p < 0.001), and the combined treatments were compared to HY-PDT alone (ǂ*p* < 0.05, ǂǂ*p* < 0.01, ǂǂǂ*p* < 0.001) and to correspondingly equal concentrations of HDACis alone (▲*p* < 0.05, ▲▲*p* < 0.01, ▲▲▲*p* < 0.001) (PPTX 82 kb).

Additional file 3: Figure S3. Effect of NaPB ± HY-PDT on DNA methylation of *CDKN1A* regulatory regions. Measurements were done in HT-29 cells after a sequential treatment starting with NaPB (1000 μM) for 24 h followed by activation with hypericin (75 nM) for 8 h. Samples treated with drug-free vehicle solvents (<0.1% DMSO) were used as the reference control. The DNA methylation levels of (A) enhancer, (B) promoter, and (C) gene body regions in the *CDKN1A* gene were analyzed and are expressed for each CpG site as the mean ± SD of three independent experiments each done in triplicates. Methylation values of 0% were set as 1% for graphical visibility (PPTX 44 kb).

## Abbreviations

CDKN1A: Cyclin-dependent kinase inhibitor 1A
ChIP: Chromatin immunoprecipitation
CRC: Colorectal cancer
EZH2: Enhancer of zeste 2 polycomb repressive complex 2 subunit
FDA: US Food and Drug Administration
H3K27ac: Histone 3 lysine 27 acetylation
H3K27me3: Histone 3 trimethylation at lysine 27
H3K4me1: Histone 3 lysine 4 monomethylation
H3K4me3: Histone 3 trimethylation at lysine 4
HBSS: Hank’s balanced salt solution
HDAC: Histone deacetylase
HDACi: Histone deacetylase inhibitor
HY: Hypericin
HY-PDT: Hypericin-mediated photodynamic therapy
mRNA: messenger RNA
MTT: 3-[4,5-Dimethylthiazol-2-yl]-2,5-diphenyltetrazolium bromide
NaPB: Sodium phenylbutyrate
PDT: Photodynamic therapy
PI: Propidium iodide
SDS: Sodium dodecyl sulfate
TMRE: Tetramethylrhodamine ethyl ester
Tsa: Trichostatin A
UVA: Ultraviolet A
Vpa: Valproic acid
