# Supplementary material for: Histone deacetylase inhibitors potentiate photodynamic therapy in colon cancer cells marked by chromatin-mediated epigenetic regulation of CDKN1A
Source: Clin Epigenetics. 2017 Jun 8;9:62. doi: 10.1186/s13148-017-0359-x (PMC5465463; doi:10.1186/s13148-017-0359-x)
Supplement: Supplementary file 1 — Mitochondrial membrane dissipation potential by HDACis ± HY-PDT. Mitochondrial membrane dissipation (TMRE+) was measured in HT-29 cells after a sequential treatment starting with HDACis (A) Saha, (B) Tsa, (C) Vpa, and (D) NaPB (for 24 h) followed by activation with hypericin (for 24 or 48 h, as indicated). Samples treated with drug-free vehicle solvents (<0.1% DMSO) were used as the reference control. The results are expressed as the percentage of control and represent the average ± SD of four independent experiments each done in singlets. Data was analyzed using one-way ANOVA with the Tukey post hoc test. All conditions were compared to the reference control (*p < 0.05, **p < 0.01, ***p < 0.001), and the combined treatments were compared to HY-PDT alone (ǂp < 0.05, ǂǂp < 0.01, ǂǂǂp < 0.001) and to correspondingly equal concentrations of HDACis alone (▲p < 0.05, ▲▲p < 0.01, ▲▲▲p < 0.001) (PPTX 55 kb). [file 13148_2017_359_MOESM1_ESM.pptx]

## Slide 1
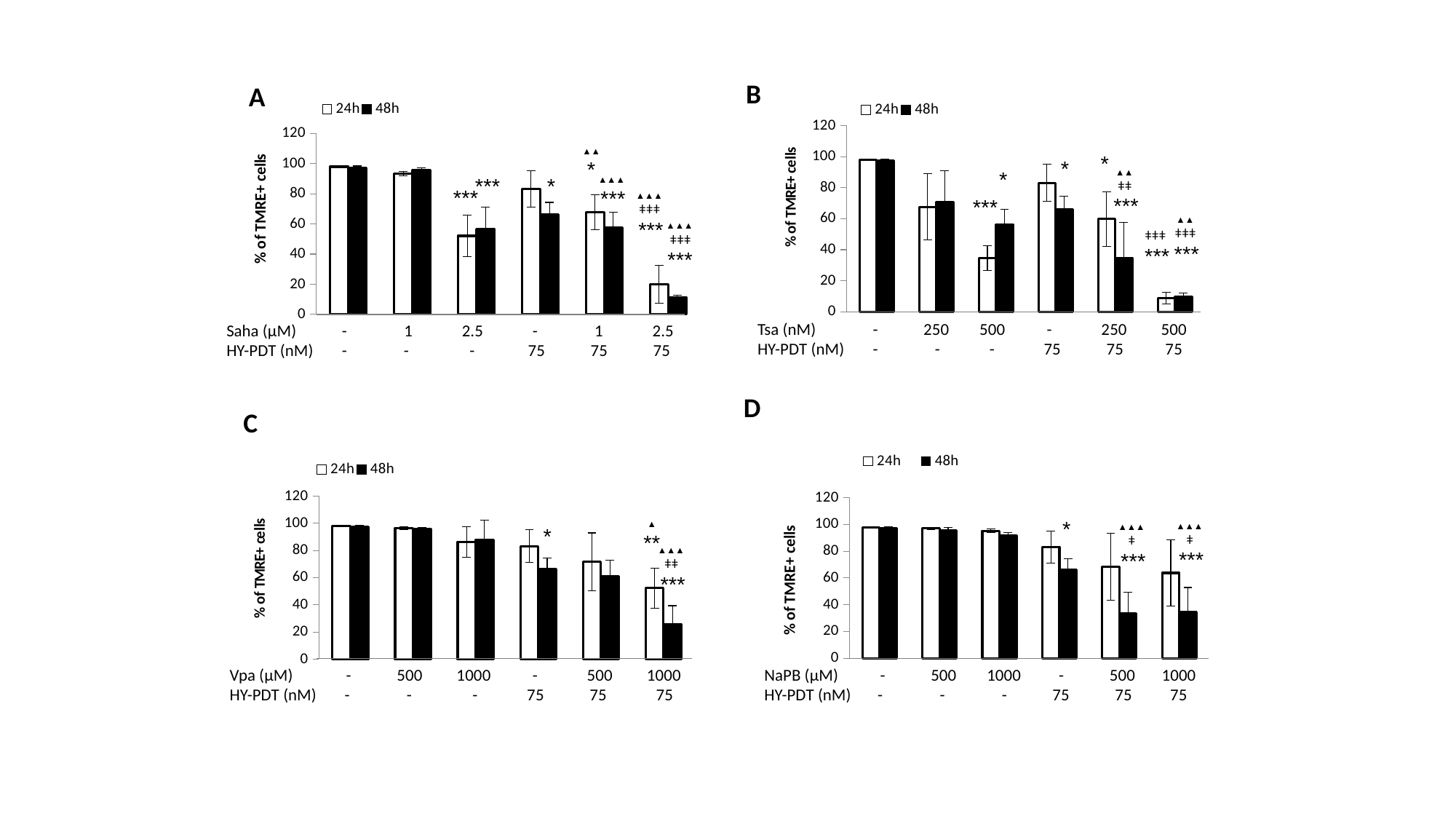

B
A
### Chart
| Category | | |
|---|---|---|
| Ct | 97.9475 | 97.42000000000002 |
| Saha 1 | 93.4525 | 95.82999999999998 |
| Saha 2 | 52.0725 | 56.4 |
| HY-PDT | 83.1325 | 66.11 |
| Saha 1 + HY-PDT | 67.61 | 57.5925 |
| Saha 2 + HY-PDT | 19.97 | 11.0375 |▲▲
*
***
*
▲▲▲
***
***
▲▲▲
ǂǂǂ
***
 ▲▲▲
 ǂǂǂ
***
### Chart
| Category | | |
|---|---|---|
| Ct | 97.9475 | 97.42000000000002 |
| Tsa 1 | 67.58500000000001 | 70.74666666666667 |
| Tsa 2 | 34.585 | 56.17 |
| HY-PDT | 83.1325 | 66.11 |
| Tsa 1 + HY-PDT | 59.775 | 34.6725 |
| Tsa 2 + HY-PDT | 8.7 | 9.565 |*
*
▲▲
ǂǂ
***
*
***
▲▲
ǂǂǂ
***
ǂǂǂ
***
Tsa (nM) - 250 500 - 250 500
HY-PDT (nM) - - - 75 75 75
Saha (µM) - 1 2.5 - 1 2.5
HY-PDT (nM) - - - 75 75 75
D
C
### Chart
| Category | | |
|---|---|---|
| Ct | 97.9475 | 97.42000000000002 |
| NaPB 1 | 97.05 | 95.64499999999998 |
| NaPB 2 | 95.2375 | 91.7875 |
| HY-PDT | 83.1325 | 66.11 |
| NaPB 1 + HY-PDT | 68.3475 | 33.5575 |
| NaPB 2 + HY-PDT | 63.765 | 34.39 |*
▲▲▲
ǂ
***
▲▲▲
ǂ
***
### Chart
| Category | | |
|---|---|---|
| Ct | 97.9475 | 97.42000000000002 |
| Vpa 1 | 96.42250000000001 | 96.0575 |
| Vpa 2 | 86.4 | 87.8 |
| HY-PDT | 83.1325 | 66.11 |
| Vpa 1 + HY-PDT | 71.58000000000001 | 60.84 |
| Vpa 2 + HY-PDT | 52.355000000000004 | 25.456666666666667 |▲
**
*
▲▲▲
ǂǂ
***
Vpa (µM) - 500 1000 - 500 1000
HY-PDT (nM) - - - 75 75 75
NaPB (µM) - 500 1000 - 500 1000
HY-PDT (nM) - - - 75 75 75
