# Supplementary material for: Histone deacetylase inhibitors potentiate photodynamic therapy in colon cancer cells marked by chromatin-mediated epigenetic regulation of CDKN1A
Source: Clin Epigenetics. 2017 Jun 8;9:62. doi: 10.1186/s13148-017-0359-x (PMC5465463; doi:10.1186/s13148-017-0359-x)
Supplement: Supplementary file 2 — Effect of HDACis ± HY-PDT on DNA and histone methyltransferase expression and DNA damage signaling. Measurements were performed in HT-29 cells after a sequential treatment starting with HDACis for 24 h followed by activation with hypericin for 8 h (A), 0–1 h (B), or 24 h (C), as indicated. The 0 h time point indicates that measurements were done immediately after hypericin activation. Samples treated with drug-free vehicle solvents (<0.1% DMSO) were used as the reference control (Ct). (A) mRNA (by qRT-PCR) levels are expressed as fold of Ct and represent the average ± SD of three independent experiments each done in triplicates. DNMT1, DNMT3A, and EZH2 mRNA levels were normalized relative to those of the housekeeping gene, GAPDH. (B) Total reactive oxygen species (ROS) and (C) histone H2AX phosphorylation levels represent the average ± SD of three independent experiments each done in singlets. H2O2 (2 M) was used as a positive treatment control for DNA damage mediated by H2AX phosphorylation (C). Phosphorylated H2AX protein levels (by FACS) were normalized relative to those of isotype H2AX. Data was analyzed using one-way ANOVA with the Tukey post hoc test and Dunnett’s multiple comparison test. All conditions were compared to the reference Ct (*p < 0.05, **p < 0.01, ***p < 0.001), and the combined treatments were compared to HY-PDT alone (ǂp < 0.05, ǂǂp < 0.01, ǂǂǂp < 0.001) and to correspondingly equal concentrations of HDACis alone (▲p < 0.05, ▲▲p < 0.01, ▲▲▲p < 0.001) (PPTX 82 kb). [file 13148_2017_359_MOESM2_ESM.pptx]

## Slide 1
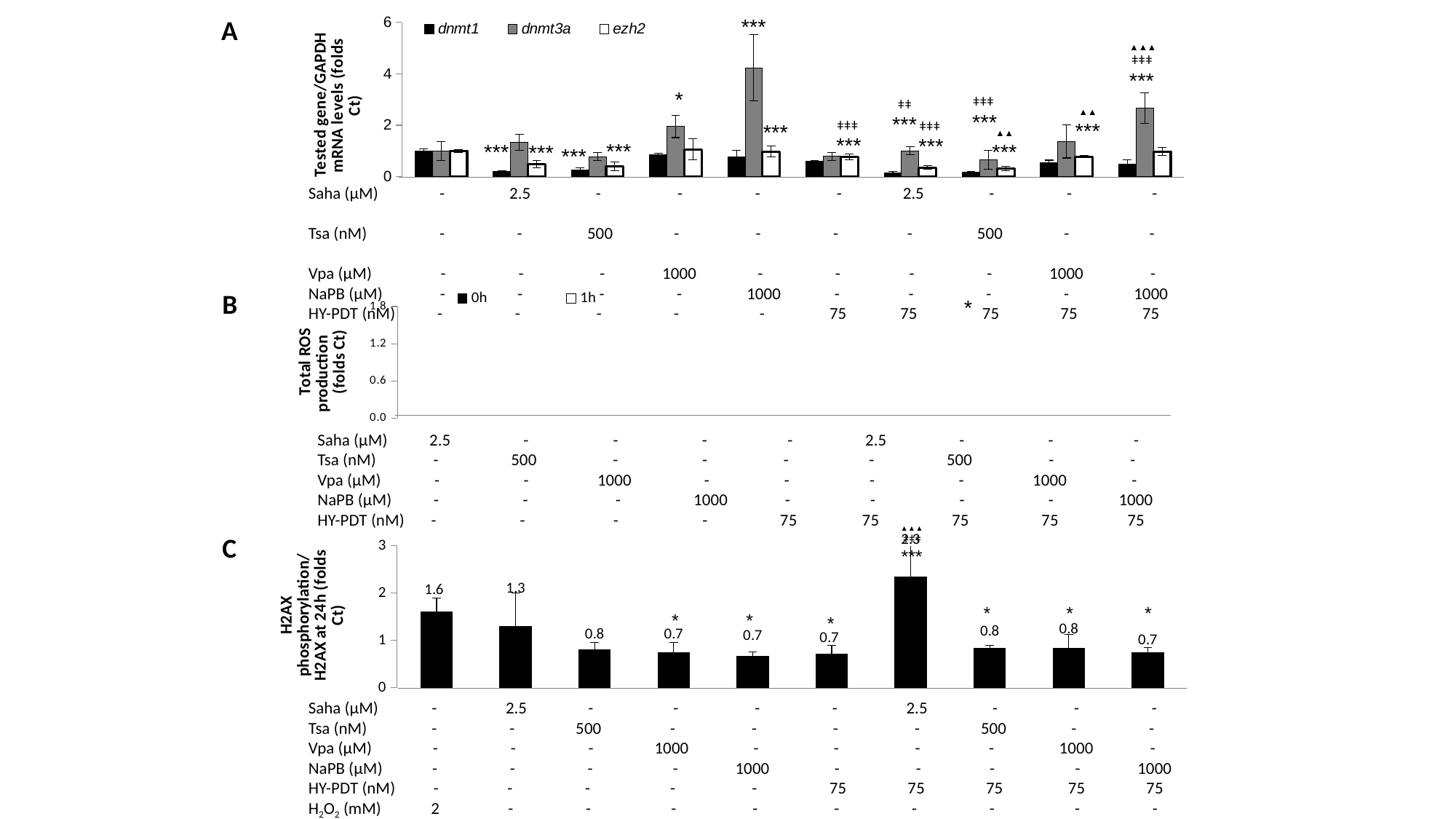

A
***
 ▲▲▲
 ǂǂǂ
***
*
ǂǂǂ
***
ǂǂ
***
▲▲
***
ǂǂǂ
***
ǂǂǂ
***
***
▲▲
***
***
***
***
***
### Chart
| Category | dnmt1 | dnmt3a | ezh2 |
|---|---|---|---|
| Ct | 1.0 | 1.0 | 1.0 |
| Saha | 0.19631482115815385 | 1.3390168359247012 | 0.49572933551500514 |
| Tsa | 0.25531622048985775 | 0.7788099960965992 | 0.39425930433789086 |
| Vpa | 0.8508014171008311 | 1.9453134728106984 | 1.0596851193797028 |
| NaPB | 0.7761240838355825 | 4.233238346320129 | 0.9750550944777787 |
| HY-PDT | 0.5908555499020098 | 0.7839290975737111 | 0.7710057674783034 |
| Saha + HY-PDT | 0.15703090972607095 | 1.008408410456518 | 0.35569461520251555 |
| Tsa + HY-PDT | 0.17953382005551982 | 0.6459507153467989 | 0.30883986153458 |
| Vpa + HY-PDT | 0.5352508249749283 | 1.3661226037446246 | 0.7786395556245506 |
| NaPB + HY-PDT | 0.4826054568500915 | 2.668639359444763 | 0.9746856193354992 |Saha (µM) - 2.5 - - - - 2.5 - - -
Tsa (nM) - - 500 - - - - 500 - -
Vpa (µM) - - - 1000 - - - - 1000 -
NaPB (µM) - - - - 1000 - - - - 1000
HY-PDT (nM) - - - - - 75 75 75 75 75
B
### Chart
| Category | 0h | 1h |
|---|---|---|
| Saha | 0.8230160894464139 | 1.039029806132035 |
| Tsa | 0.9520043632397054 | 1.0614911606456572 |
| Vpa | 0.8428551949822743 | 0.9437185071312665 |
| NaPB | 0.7774747750204526 | 0.8962336664104534 |
| HY-PDT | 0.9622988819198254 | 1.0203262447689811 |
| Saha + HY-PDT | 1.034701390782656 | 1.1026133743274404 |
| Tsa + HY-PDT | 1.1056722116171256 | 1.3586130327098813 |
| Vpa + HY-PDT | 0.7901554404145076 | 0.9650696045776753 |
| NaPB + HY-PDT | 0.8187210253613307 | 0.9473908958920486 |*
Saha (µM) 2.5 - - - - 2.5 - - -
Tsa (nM) - 500 - - - - 500 - -
Vpa (µM) - - 1000 - - - - 1000 -
NaPB (µM) - - - 1000 - - - - 1000
HY-PDT (nM) - - - - 75 75 75 75 75
▲▲▲
 ǂǂǂ
***
*
*
*
*
*
*
### Chart
| Category | |
|---|---|
| Ct + (H2O2 2M) | 1.6064906332729787 |
| Saha | 1.293916990993531 |
| Tsa | 0.8023350983106827 |
| Vpa | 0.7458535530590931 |
| NaPB | 0.6558229359406762 |
| HY | 0.7112789161382657 |
| Saha + HY-PDT | 2.348752514214167 |
| Tsa + HY-PDT | 0.8394338019632607 |
| Vpa + HY-PDT | 0.8330949998879724 |
| NaPB + HY-PDT | 0.7427894170776348 |Saha (µM) - 2.5 - - - - 2.5 - - -
Tsa (nM) - - 500 - - - - 500 - -
Vpa (µM) - - - 1000 - - - - 1000 -
NaPB (µM) - - - - 1000 - - - - 1000
HY-PDT (nM) - - - - - 75 75 75 75 75
H2O2 (mM) 2 - - - - - - - - -
C
