# Supplementary material for: Histone deacetylase inhibitors potentiate photodynamic therapy in colon cancer cells marked by chromatin-mediated epigenetic regulation of CDKN1A
Source: Clin Epigenetics. 2017 Jun 8;9:62. doi: 10.1186/s13148-017-0359-x (PMC5465463; doi:10.1186/s13148-017-0359-x)
Supplement: Supplementary file 3 — Effect of NaPB ± HY-PDT on DNA methylation of CDKN1A regulatory regions. Measurements were done in HT-29 cells after a sequential treatment starting with NaPB (1000 μM) for 24 h followed by activation with hypericin (75 nM) for 8 h. Samples treated with drug-free vehicle solvents (<0.1% DMSO) were used as the reference control. The DNA methylation levels of (A) enhancer, (B) promoter, and (C) gene body regions in the CDKN1A gene were analyzed and are expressed for each CpG site as the mean ± SD of three independent experiments each done in triplicates. Methylation values of 0% were set as 1% for graphical visibility (PPTX 44 kb). [file 13148_2017_359_MOESM3_ESM.pptx]

## Slide 1
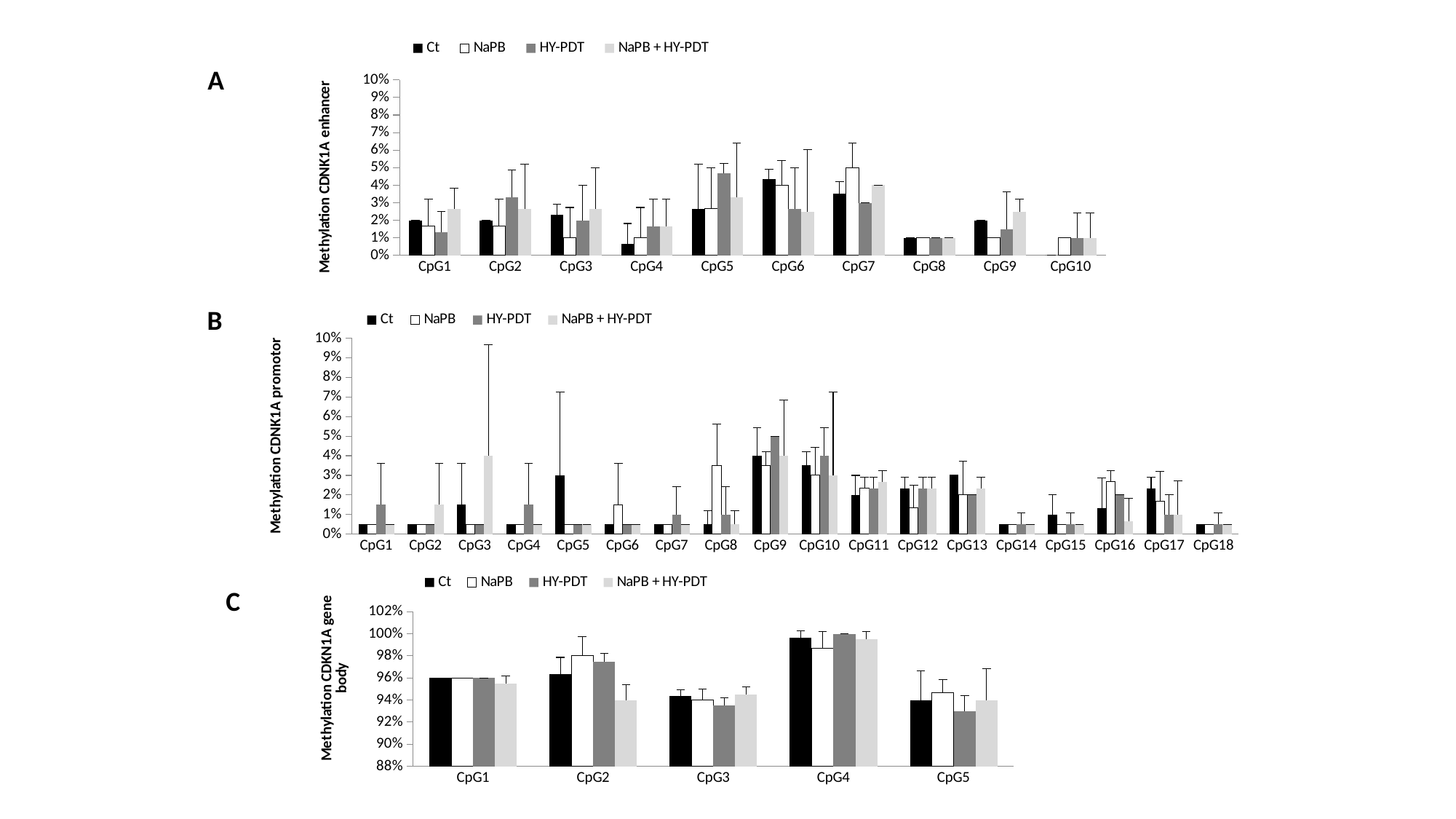

### Chart
| Category | Ct | NaPB | HY-PDT | NaPB + HY-PDT |
|---|---|---|---|---|
| CpG1 | 0.02 | 0.016666666666666666 | 0.013333333333333334 | 0.02666666666666667 |
| CpG2 | 0.02 | 0.016666666666666666 | 0.03333333333333333 | 0.02666666666666667 |
| CpG3 | 0.023333333333333334 | 0.01 | 0.02 | 0.02666666666666667 |
| CpG4 | 0.006666666666666667 | 0.01 | 0.016666666666666666 | 0.016666666666666666 |
| CpG5 | 0.02666666666666667 | 0.02666666666666667 | 0.04666666666666667 | 0.03333333333333333 |
| CpG6 | 0.043333333333333335 | 0.04 | 0.02666666666666667 | 0.025 |
| CpG7 | 0.035 | 0.05 | 0.03 | 0.04 |
| CpG8 | 0.01 | 0.01 | 0.01 | 0.01 |
| CpG9 | 0.02 | 0.01 | 0.015 | 0.025 |
| CpG10 | 0.0 | 0.01 | 0.01 | 0.01 |A
### Chart
| Category | Ct | NaPB | HY-PDT | NaPB + HY-PDT |
|---|---|---|---|---|
| CpG1 | 0.005 | 0.005 | 0.015 | 0.005 |
| CpG2 | 0.005 | 0.005 | 0.005 | 0.015 |
| CpG3 | 0.015 | 0.005 | 0.005 | 0.04 |
| CpG4 | 0.005 | 0.005 | 0.015 | 0.005 |
| CpG5 | 0.03 | 0.005 | 0.005 | 0.005 |
| CpG6 | 0.005 | 0.015 | 0.005 | 0.005 |
| CpG7 | 0.005 | 0.005 | 0.01 | 0.005 |
| CpG8 | 0.005 | 0.035 | 0.01 | 0.005 |
| CpG9 | 0.04 | 0.035 | 0.05 | 0.04 |
| CpG10 | 0.035 | 0.03 | 0.04 | 0.03 |
| CpG11 | 0.02 | 0.023333333333333334 | 0.023333333333333334 | 0.02666666666666667 |
| CpG12 | 0.023333333333333334 | 0.013333333333333334 | 0.023333333333333334 | 0.023333333333333334 |
| CpG13 | 0.03 | 0.02 | 0.02 | 0.023333333333333334 |
| CpG14 | 0.005 | 0.005 | 0.005 | 0.005 |
| CpG15 | 0.01 | 0.005 | 0.005 | 0.005 |
| CpG16 | 0.013333333333333334 | 0.02666666666666667 | 0.02 | 0.006666666666666667 |
| CpG17 | 0.023333333333333334 | 0.016666666666666666 | 0.01 | 0.01 |
| CpG18 | 0.005 | 0.005 | 0.005 | 0.005 |B
### Chart
| Category | Ct | NaPB | HY-PDT | NaPB + HY-PDT |
|---|---|---|---|---|
| CpG1 | 0.96 | 0.96 | 0.96 | 0.955 |
| CpG2 | 0.9633333333333333 | 0.98 | 0.975 | 0.94 |
| CpG3 | 0.9433333333333334 | 0.9400000000000001 | 0.935 | 0.945 |
| CpG4 | 0.9966666666666667 | 0.9866666666666667 | 1.0 | 0.995 |
| CpG5 | 0.9400000000000001 | 0.9466666666666667 | 0.9299999999999999 | 0.94 |C
